# What Factors Influence the Perception of Longevity?

**DOI:** 10.1093/geroni/igaa057.1460

**Published:** 2020-12-16

**Authors:** Camela Barker, Laura Lerebours, Benjamin Gary, Olivia Harper, Devyn Dixon, Tyson Brown, Roland Thorpe, Keith Whitfield

**Affiliations:** 1 Duke University, Durham, North Carolina, United States; 2 Johns Hopkins University, Baltimore, Maryland, United States; 3 Wayne State University, Detroit, Michigan, United States

## Abstract

Previous research has highlighted the link between health-related factors and longevity. In particular, indices such as poor cardiovascular reactivity, high BMI, stress, and respiratory functioning have been shown to increase risk of mortality. However, little is known about identifying specific health and psychosocial behaviors that compromise the perception of longevity in African Americans. The Study of Longevity and Stress in African Americans (SOLSAA) was designed to examine psychosocial and health factors that are characteristic of survivorship in African Americans families. Data from 570 participants in the SOLSAA were used to examine the relationship between psychosocial and health-related factors among individuals aged 50 to 99 years (M = 67.6, SD = 11.5). Perception of longevity was positively correlated with sex, r = .058, education, r = .021, diastolic blood pressure r = .067, respiratory functioning, r = .150 and body mass index, r = .027, but negatively correlated with age, r = -.205, systolic blood pressure, r = -.050, and perceived stress, r = -.106. Regression analyses revealed, not surprisingly, that as participants aged, perception of longevity decreased. Furthermore, women’s perception of longevity was greater than that of men even after controlling for age. Given the longer life expectancy for women, this was also expected. Interestingly, high levels of perceived stress predicted reduced perception of longevity. These preliminary results suggest that the perception of stress is an important psychosocial factor for older African American’s self-forecasting of their chances for longevity beyond just simply age or physical health status.

